# The biased M3 mAChR ligand PD 102807 mediates qualitatively distinct signaling to regulate airway smooth muscle phenotype

**DOI:** 10.1016/j.jbc.2023.105209

**Published:** 2023-09-01

**Authors:** Eric Tompkins, Bogdana Mimic, Raymond B. Penn, Tonio Pera

**Affiliations:** Department of Medicine, Center for Translational Medicine, Jane and Leonard Korman Respiratory Institute, Philadelphia, Pennsylvania, USA

**Keywords:** asthma, airway, airway smooth muscle, G protein-coupled receptor, biased receptor pharmacology

## Abstract

Airway smooth muscle (ASM) cells attain a hypercontractile phenotype during obstructive airway diseases. We recently identified a biased M3 muscarinic acetylcholine receptor (mAChR) ligand, PD 102807, that induces GRK-/arrestin-dependent AMP-activated protein kinase (AMPK) activation to inhibit transforming growth factor-β–induced hypercontractile ASM phenotype. Conversely, the balanced mAChR agonist, methacholine (MCh), activates AMPK yet does not regulate ASM phenotype. In the current study, we demonstrate that PD 102807- and MCh-induced AMPK activation both depend on Ca^2+^/calmodulin-dependent kinase kinases (CaMKKs). However, MCh-induced AMPK activation is calcium-dependent and mediated by CaMKK1 and CaMKK2 isoforms. In contrast, PD 102807-induced signaling is calcium-independent and mediated by the atypical subtype protein kinase C-iota and the CaMKK1 (but not CaMKK2) isoform. Both MCh- and PD 102807-induced AMPK activation involve the AMPK α1 isoform. PD 102807-induced AMPK α1 (but not AMPK α2) isoform activation mediates inhibition of the mammalian target of rapamycin complex 1 (mTORC1) in ASM cells, as demonstrated by increased Raptor (regulatory-associated protein of mTOR) phosphorylation as well as inhibition of phospho-S6 protein and serum response element–luciferase activity. The mTORC1 inhibitor rapamycin and the AMPK activator metformin both mimic the ability of PD 102807 to attenuate transforming growth factor-β–induced α-smooth muscle actin expression (a marker of hypercontractile ASM). These data indicate that PD 102807 transduces a signaling pathway (AMPK-mediated mTORC1 inhibition) qualitatively distinct from canonical M3 mAChR signaling to prevent pathogenic remodeling of ASM, thus demonstrating PD 102807 is a biased M3 mAChR ligand with therapeutic potential for the management of obstructive airway disease.

M3 muscarinic acetylcholine receptors (mAChRs) are crucial regulators of airway smooth muscle (ASM) tone. Increased M3 mAChR signaling is also a key driver of pathogenesis in asthma and COPD, including increased ASM tone and airway obstruction (1), inflammation (2, 3, 4), and airway remodeling (2, 4, 5). The effectiveness of inhaled anticholinergics (mAChR antagonists) in reversing bronchoconstriction and airway obstruction provides strong evidence that increased canonical M3 mAChR signaling is pathogenic in obstructive airways diseases. Characterizing signaling and functional effects of novel mAChR ligands is clinically beneficial considering the critical role of mAChRs in the physiological and pathological regulation of airway tone. Acetylcholine is an endogenous mAChR agonist that activates M3 mAChRs on ASM cells. Canonical M3 mAChR signaling is mediated by Gq subunits of heterotrimeric G proteins and leads to calcium mobilization that promotes ASM contraction and airway obstruction. Coincidently, agonist-bound receptor is phosphorylated by GRKs followed by recruitment of scaffold proteins arrestins to the phosphorylated receptors initiating the process of receptor desensitization and internalization (6, 7). Previous studies indicate that arrestins induce selective signaling independent of canonical G-protein signaling (8, 9). Since the discovery of GPCRs, ligands typically have been classified based on their ability to bind the receptor and promote second messenger production. A more recent and growing body of evidence now suggests a much more nuanced regulation of GPCR signaling. Receptors are not only turned on or off with respect to their second messenger production but they may be fine-tuned to induce signaling qualitatively distinct from second messenger production: qualitative or biased signaling. Biased signaling is increasingly being identified for a variety of GPCRs (10), and ligands can be classified based on preferential activation of G protein- or GRK/arrestin-mediated signaling. Biased GPCR signaling has been implicated as an important regulator of the pathophysiology of different human diseases. For example, β-arrestin-biased signaling by the beta-adrenoceptor may be beneficial for heart function (11, 12). Conversely, a number of studies have suggested that beta-2-AR arrestin-biased signaling is pathogenic in the lung, promoting an asthmatic phenotype, including mucous metaplasia and airway hyperresponsiveness (13, 14, 15, 16). Recent studies have identified G protein biased beta-2-AR ligands that may be therapeutically beneficial in asthma (17, 18, 19). These studies collectively highlight the potential clinical utility of biased GPCR ligands in human diseases.

We recently identified PD 102807—an established M4 mAChR antagonist (20, 21)—as a biased M3 mAChR ligand (22). In M3 mAChR-expressing ASM cells, PD 102807 promotes arrestin recruitment to the M3 mAChR and GRK-dependent signaling with no effect on Gq-mediated activation of calcium signaling. Most importantly, PD 102807 treatment results in inhibition of transforming growth factor-β (TGF-β)-induced ASM phenotype modulation and hypercontractility. This is in contrast to an unbiased or balanced M3 mAChR agonist that does not inhibit the development of ASM hypercontractile phenotype. We also identified AMP-activated protein kinase (AMPK) as a major effector activated by PD 102807-induced signaling. However, the molecular mechanisms involved in AMPK activation and downstream regulation of ASM cell phenotype are not established. AMPK is an important regulator of a wide variety of cell functions, and it can be activated by changes in cytosolic AMP/ATP ratio (23), as well as by various kinases, including serine/threonine kinase 11 (STK11, also known as liver kinase B1 (LKB1)), TGF-β-activated kinase (TAK1), and calcium/calmodulin-dependent kinase kinase (CaMKK) (24). Therefore, in the present study, we aimed at establishing the mechanisms involved in biased ligand PD 102807- and unbiased methacholine (MCh)-induced activation of AMPK signaling and phenotype modulation in ASM cells.

## Results

### M3 mAChR-induced AMPK activation is mediated by CaMKKs

**Figure 1:**
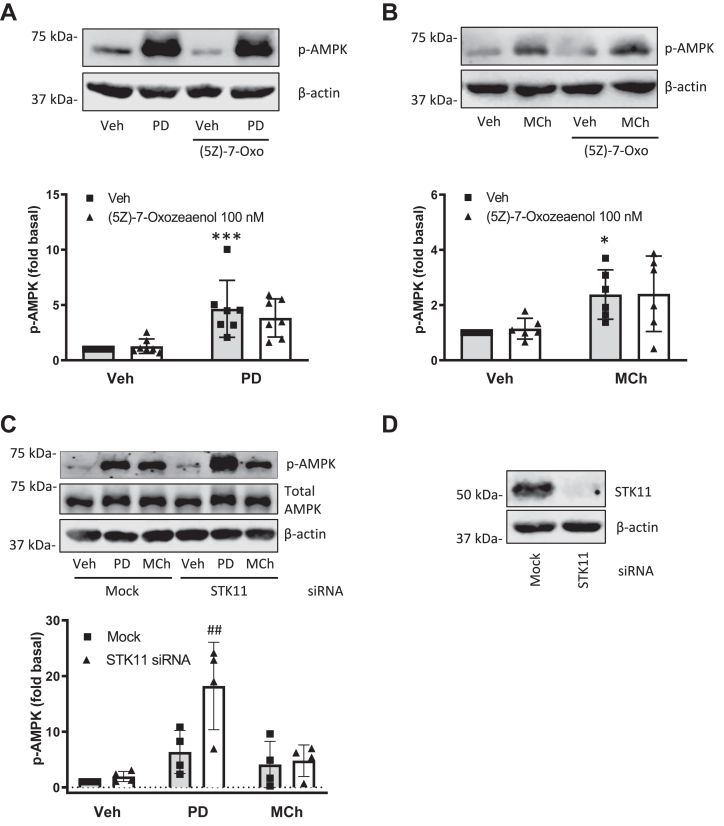
**AMPK kinases TAK1 and STK11 (LKB1) do not mediate M3 mAChR-induced AMPK phosphorylation.***A* and *B*, cells were preincubated with 5z-7-oxozeaenol (100 nM) for 15 min then stimulated with (*A*) PD 102807 (100 μM; t = 20 min) or (*B*) MCh (100 μM; t = 5 min). *C*, following siRNA transfection against STK11 (as described in Experimental procedures), cells were stimulated with PD 102807 or MCh. *D*, STK11 knockdown was confirmed by immunoblotting. Representative blots are shown. Loading was corrected for β-actin or total AMPK. Data are means ± SD. ∗*p* < 0.05, ∗∗∗ *p* < 0.001 *versus* vehicle stimulation; ##*p* < 0.01 *versus* mock-transfected PD 102807; one-way ANOVA followed by Bonferroni multiple comparison test. AMPK, AMP-activated protein kinase; LKB1, liver kinase B1; mAChR, muscarinic acetylcholine receptor; MCh, methacholine.

**Figure 2:**
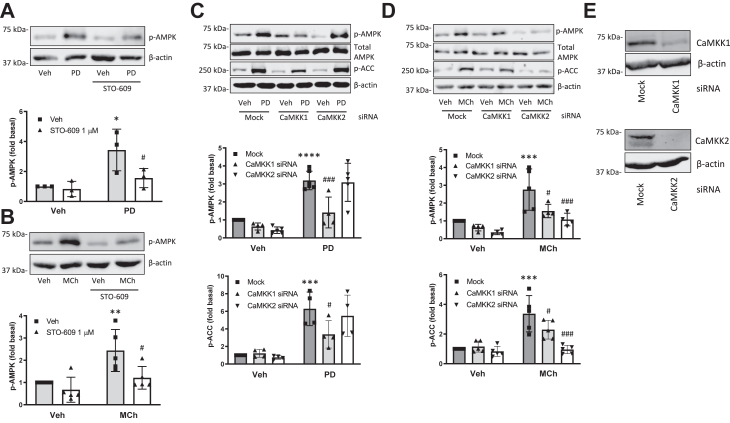
**CaMKKs mediate M3 mAChR-induced AMPK phosphorylation.***A* and *B*, cells were preincubated with STO-609 (1 μM) for 15 min and then stimulated with (*A*) PD 102807 (100 μM; t = 20 min) or (*B*) MCh (100 μM; t = 5 min). *C* and *D*, cells were transfected with siRNA against CaMKK1 or CaMKK2 and then stimulated with (*C*) PD 102807 or (*D*) MCh. AMPK and ACC phosphorylation was determined using phospho-specific antibodies. *E*, knockdown of CaMKK isoforms was confirmed by immunoblotting. Representative blots are shown. Loading was corrected for total AMPK or β-actin. Data are means ± SD. ∗*p* < 0.05, ∗∗*p* < 0.01, ∗∗∗ *p* < 0.001, ∗∗∗∗*p* < 0.0001 *versus* vehicle stimulation, #*p* < 0.05, ###*p* < 0.001 *versus* respective mock-transfected PD 102807 or MCh; one-way ANOVA followed by Bonferroni multiple comparison test. ACC, acetyl-CoA carboxylase; AMPK, AMP-activated protein kinase; CaMKKs, Ca^2+^/calmodulin-dependent kinase kinases; mAChR, muscarinic acetylcholine receptor; MCh, methacholine.

We previously demonstrated that stimulation of human ASM cells with M3 mAChR ligands results in AMPK activation *via* either canonical (Gq-dependent) or biased (GRK/arrestin-dependent) signaling pathways (22). In order to further delineate the mechanism of M3-mediated AMPK activation, we focused on known kinases upstream of AMPK: TAK1, STK11 (LKB1), and CaMKK. Pretreatment of human ASM cells with the small molecule inhibitor of TAK1, 5z-7-oxozeaenol (Fig. 1, *A* and *B*) or small interfering RNA (siRNA)-mediated knockdown (STK11; Fig. 1*C*) did not decrease M3-mediated AMPK phosphorylation induced by PD 102807 or MCh. Interestingly, siRNA-mediated knockdown of STK11 led to an 80% increase of PD 102807-induced phospho-AMPK (p-AMPK) signal. To assess the role of CaMKK in M3-mediated phosphorylation of AMPK, we used the small molecule inhibitor of CaMKK, STO-609. Pretreatment of human ASM cells with STO-609 inhibited both PD 102807- and MCh-induced p-AMPK, by ∼77% and ∼83%, respectively (Fig. 2, *A* and *B*). To assess the role of CaMKK isoforms in M3-mediated AMPK phosphorylation, we used siRNA to selectively knock down either CaMKK [CaMKK1 (aka CaMKKα) or CaMKK2 (aka CaMKKβ)] and assessed phosphorylation of AMPK as well as phosphorylation of the AMPK substrate acetyl-CoA carboxylase (ACC), as a measure of AMPK activation. Knockdown of CaMKK1 inhibited PD 102807-induced phosphorylation of AMPK by ∼81% and its downstream target ACC by ∼50% (Fig. 2*C*). Knockdown of CaMKK2 did not affect PD 102807-induced activation of AMPK signaling. Knockdown of either isoform reduced the MCh-induced p-AMPK and p-ACC (Fig. 2*D*), although knockdown of CaMKK2 was more effective at abrogating MCh-induced p-AMPK (∼96% inhibition) compared to CaMKK1 knockdown (∼68% inhibition). Similarly, MCh-induced p-ACC was fully inhibited by CaMKK2 knockdown, whereas CaMKK1 knockdown resulted in a ∼50% inhibition (Fig. 2*D*). These findings suggest CaMKK isoforms play a major role in M3-mediated AMPK activation, whereas there is no discernible role for STK11/LKB1 or TAK1. Further, biased ligand PD 102807 differs from balanced ligand MCh in terms of CaMKK-mediated activation of AMPK signaling in ASM cells.

### PD 102807-induced AMPK phosphorylation is calcium independent

**Figure 3:**
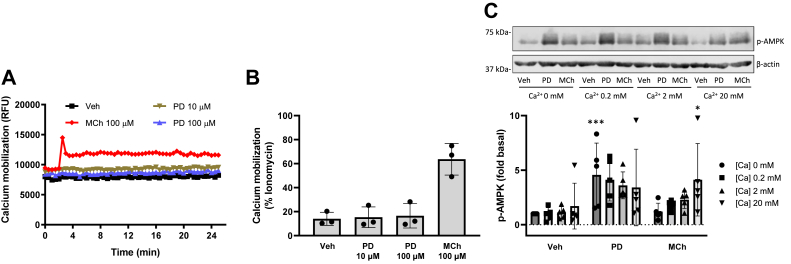
**PD 102807-induced p-AMPK is independent of calcium mobilization.***A* and *B*, cells were stimulated with PD 102807 (10 and 100 μM) or MCh (100 μM), and calcium mobilization was measured using fluo-4 AM calcium indicator and Flexstation III as described in Experimental procedures. *C*, cells were permeabilized using β-escin (100 nM) then treated with increasing concentrations of CaCl_2_ (0, 0.2, 2, and 20 mM) and allowed to equilibrate for 5 min. Cells were then stimulated with PD 102807 (100 μM; t = 20 min) or MCh (100 μM; t = 5 min). Cells were lysed, and phosphorylation status of AMPK was determined by immunoblotting. Representative blots are shown. Loading was corrected for β-actin. Data are means ± SD. ∗*p* < 0.05, ∗∗∗*p* < 0.001 *versus* vehicle stimulation; one-way ANOVA followed by Bonferroni multiple comparison test. AMPK, AMP-activated protein kinase; MCh, methacholine; p-AMPK, phospho-AMPK.

Our previous studies have shown that PD 102807 and MCh both induce AMPK phosphorylation, albeit *via* different mechanisms and with different kinetic profiles. While the MCh-induced p-AMPK is mediated by Gq, the PD 102807-induced p-AMPK is mediated by GRK/arrestin (22). In addition, PD 102807 induces sustained AMPK phosphorylation, whereas MCh-induced AMPK phosphorylation wanes in a matter of minutes. Since CaMKK signaling is generally associated with calcium mobilization, we sought to determine whether calcium mobilization plays any role in PD 102807-induced AMPK phosphorylation. We previously determined that PD 102807 does not induce calcium mobilization, but our measurements were limited to acute (70 s) stimulation. To clarify potential changes throughout the duration of PD 102807 stimulation of cells, we measured intracellular calcium concentrations up to 25 min. Consistent with our previous study, PD 102807 did not induce calcium mobilization at any point during the stimulation (Fig. 3, *A* and *B*).

In order to determine whether AMPK activation requires intracellular calcium, we permeabilized the cells with β-escin and clamped the calcium concentrations at 0, 0.2, 2, and 20 mM. Cells were then stimulated with PD 102807 or MCh to determine the magnitude of p-AMPK induction at each calcium concentration. Under calcium-free conditions ([Ca^2+^] 0 mM), PD 102807 induced a 4.5-fold increase of AMPK phosphorylation. With [Ca^2+^] clamped at 0.2, 2, or 20 mM, PD 102807 induced approximately a 3.5- to 4-fold increase of p-AMPK. Under calcium-free conditions ([Ca^2+^] 0 mM), MCh failed to induce p-AMPK whereas increasing calcium concentrations led to increases in MCh-induced p-AMPK; 0.2, 2, and 20 mM calcium resulted in a 1.5-, 2-, and 5-fold increase of p-AMPK, respectively (Fig. 3*C*). Collectively, these data indicate that PD 102807-induced p-AMPK is independent of calcium, whereas MCh-induced p-AMPK requires calcium.

### The atypical protein kinase C iota is required for PD 102807-induced AMPK phosphorylation

**Figure 4:**
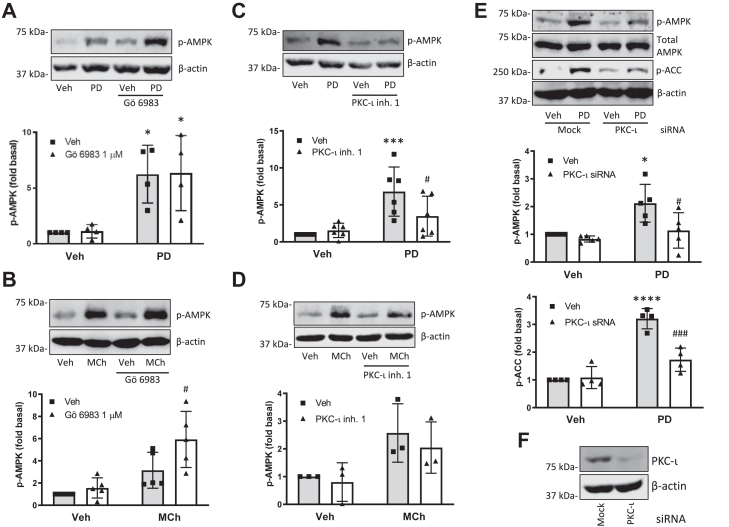
**PD 102807 induces p-AMPK in a PKC-iota-dependent manner.***A* and *B*, cells were stimulated with (*A*) PD 102807 (100 μM; t = 20 min) or (*B*) MCh (100 μM; t = 5 min) in the presence or absence of the pan-PKC inhibitor Gö 6983 (1 μM; 15 min preincubation). *C* and *D*, cells were stimulated with (*C*) PD 102807 or (*D*) MCh in the presence or absence of the selective small molecule PKC-ι inhibitor 1 (10 μM; 15 min preincubation). Phosphorylation status of AMPK was determined using a phospho-specific AMPK antibody. *E*, cells were transfected with siRNA against PKC-ι and subsequently stimulated with PD 102807. Phosphorylation status of AMPK and ACC was determined using phospho-specific antibodies. *F*, PKC-ι knockdown was confirmed by immunoblotting. Representative blots are shown. Loading was corrected for β-actin or total AMPK. Data are means ± SD values from 3 to five experiments. ∗*p* < 0.05, ∗∗∗ *p* < 0.001, ∗∗∗∗*p* < 0.0001 *versus* vehicle stimulation, #*p* < 0.05, ###*p* < 0.001 *versus* respective mock-transfected PD 102807 or MCh; one-way ANOVA followed by Bonferroni multiple comparison test. ACC, ACC, acetyl-CoA carboxylase; AMPK, AMP-activated protein kinase; MCh, methacholine; p-AMPK, phospho-AMPK; PKC-ι, protein kinase C iota.

The protein kinase C family members are important effectors downstream of Gq-coupled GPCRs, including the M3 mAChR (25). Protein kinases C (PKCs) are therefore important mediators of canonical Gq signaling and have also been implicated in receptor desensitization due to their ability to phosphorylate GPCRs (26). To assess whether PKCs play any role in noncanonical, biased M3 mAChR signaling, we pretreated cells with the pan-PKC inhibitor Gö 6983 (1 μM; 15 min) which inhibits conventional (calcium- and diacylglycerol-dependent) and novel (diacylglycerol-dependent) PKC isoforms, as well as the atypical (calcium- and diacylglycerol-independent) isoform PKC-ζ [but not the atypical isoform protein kinase C iota (PKC-ι)]. Gö 6983 did not affect PD 102807-induced p-AMPK, whereas it potentiated MCh-induced p-AMPK (Fig. 4, *A* and *B*). Given PD 102807 induces M3 signaling that is independent of Gq/calcium mobilization, we sought to determine if the other calcium-insensitive atypical isoform, PKC-ι, participates in PD 102807- or MCh-induced AMPK signaling. The PKC-ι inhibitor, compound 19 (aka PKC-ι inhibitor 1; 10 μM; 15 min preincubation), inhibited PD 102807-induced p-AMPK, whereas it had no effect on MCh-induced p-AMPK (Fig. 4, *C* and *D*). Similarly, siRNA knockdown of PKC-ι inhibited PD 102807-induced p-AMPK and p-ACC (Fig. 4, *E* and *F*). A known activator of PKC-ι, phosphatidylinositol 3-kinase (PI3K) (27), does not play a role in PD 102807-induced phosphorylation of AMPK, as indicated by lack of effectiveness of the PI3K inhibitors LY294002 (10 or 30 μM; 15 min preincubation) and wortmannin (30 or 100 nM; 15 min preincubation) (Fig. S1). These data suggest that biased M3 mAChR signaling by PD 102807 requires PKC-ι to activate AMPK, whereas MCh-induced M3 mAChR does not. Canonical M3 mAChR signaling requires calcium to activate CaMKK-AMPK signaling in ASM cells and is independent of conventional and novel PKCs which are generally associated with Gq signaling.

### AMPK α1 is the major isoform activated by M3 mAChR signaling

**Figure 5:**
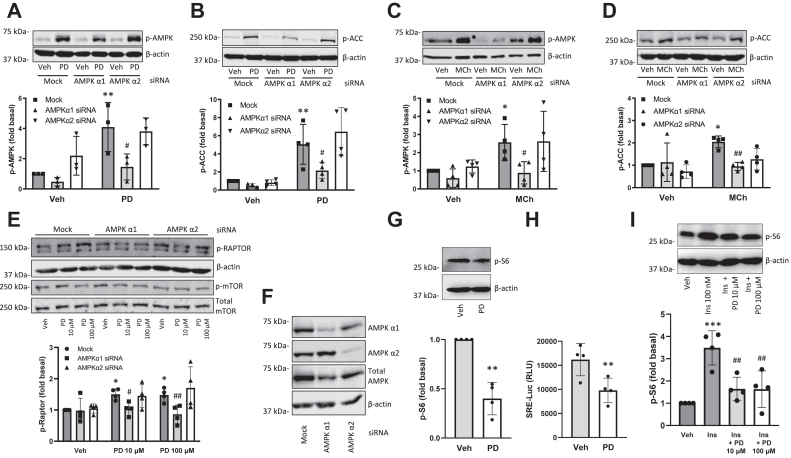
**PD 102807 induces phosphorylation of AMPK alpha 1 and Raptor to inhibit mTOR signaling in ASM cells.***A*–*D*, after siRNA transfection against AMPK α1 or α2 subunit cells were stimulated with (*A* and *B*) PD 102807 (100 μM; t = 20 min) or (*C* and *D*) MCh (100 μM; t = 5 min) and phosphorylation of (*A* and *C*) AMPK or (*B* and *D*) ACC was determined by immunoblotting using phospho-specific antibodies. *E*, after siRNA transfection against AMPK α1 or α2 subunit, cells were stimulated with PD 102807 (10 or 100 μM; t = 20 min) and expression of p-Raptor, β-actin, p-mTOR or total mTOR was determined by immunoblotting. *F*, AMPK α1 and α2 knockdown was confirmed by immunoblotting. *G*, PD 102807 (10 μM; 20 min stimulation) inhibits basal phosphorylation od S6 ribosomal protein. *H*, PD 102807 (10 μM; 18 h stimulation) inhibits basal SRE-Luc activity. *I*, PD 102807 (10 or 100 μM; 20 min pretreatment) inhibits insulin-induced (100 nM; 30 min) phosphorylation of S6 ribosomal protein. Representative blots are shown. Loading was corrected for β-actin or total AMPK. Data are means ± SD. ∗*p* < 0.05, ∗∗*p* < 0.01, ∗∗∗*p* < 0.001 *versus* vehicle stimulation, #*p* < 0.05, ##*p* < 0.01 *versus* respective vehicle pretreated, stimulated condition; one-way ANOVA followed by Bonferroni multiple comparison test; two-tailed Student’s *t* test. AMPK, AMP-activated protein kinase; ASM, Airway smooth muscle; MCh, methacholine; mTOR, the mammalian target of rapamycin; SRE-Luc, serum response element–driven luciferase activity.

Next, we assessed if differential activation of AMPK signaling by PD 102807 and MCh involves differential activation of AMPK isoforms. To determine which AMPK isoform is involved in M3-mediated signaling, we used siRNA to knock down AMPK α1 or AMPK α2 isoforms and then assessed phosphorylation of AMPK and ACC. Knockdown of AMPK α1 resulted in an ∼85% reduction of the PD 102807-induced p-AMPK (Fig. 5*A*) and complete abrogation of the MCh-induced p-AMPK (Fig. 5*C*). Alternatively, AMPK α2 knockdown had no effect on either PD 102807- or MCh-induced p-AMPK (Fig. 5, *A* and *C*). Similarly, we determined that AMPK α1 knockdown resulted in complete abrogation of both PD 102807- and MCh-induced ACC phosphorylation (Fig. 5, *B* and *D*). AMPK α2 knockdown had no effect on PD 102807-induced p-ACC (Fig. 5*B*) but trended toward inhibition of MCh-induced p-ACC (Fig. 5*D*). Together, these data indicate that AMPK α1 is the AMPK isoform phosphorylated through M3 mAChR signaling either *via* biased or balanced M3 mAChR ligands.

### PD 102807-induced AMPK α1 activation regulates ASM α-smooth muscle actin expression *via* Raptor phosphorylation

In the next set of experiments, we assessed the effect of PD 102807 on signaling downstream of AMPK. AMPK is an established regulator of mTOR pathways due to its ability to phosphorylate regulatory-associated protein of mTOR (Raptor), a major subunit of the mTOR complex 1 (mTORC1) (28). Therefore, we sought to determine a role, if any, for mTOR signaling downstream of PD 102807-induced AMPK activation. PD 102807 induced a 1.5-fold increase in Raptor phosphorylation that was abolished by AMPK α1 knockdown but not affected by AMPK α2 knockdown (Fig. 5, *E* and *F*). PD 102807 did not affect mTOR phosphorylation (Fig. 5*E*). To evaluate signaling outcomes downstream of mTOR (29), we determined phosphorylation of S6 ribosomal protein by Western blotting and activation of serum response element by luciferase activity assay. PD 102807 inhibited both basal and insulin-induced S6 ribosomal protein phosphorylation as well as serum response element (SRE)-driven luciferase activity (SRE-Luc) (Figs. 5, *G* and *H* and *I* and S2). The ability of PD 102807 to inhibit phosphorylation of S6 protein and SRE-Luc activity was impaired in ASM cells that express no or very low levels of M3 mAChR (Fig. S3). To demonstrate the robustness of our findings, we also performed experiments in HEK 293 cells which endogenously express M3 mAChR. Similar to our findings in ASM cells, in HEK 293 cells, PD 102807 induced M3 mAChR-dependent phosphorylation of AMPK, as well as inhibition of insulin-induced S6 ribosomal protein phosphorylation and basal SRE-Luc activity (Figs. S2 and S3). Collectively these data indicate that PD 102807 activates AMPK α1 to inhibit mTORC1 by phosphorylating Raptor. This results in inhibition of both translation (phosphorylation of S6 ribosomal protein) and transcription (SRE-Luc) pathways.

**Figure 6:**
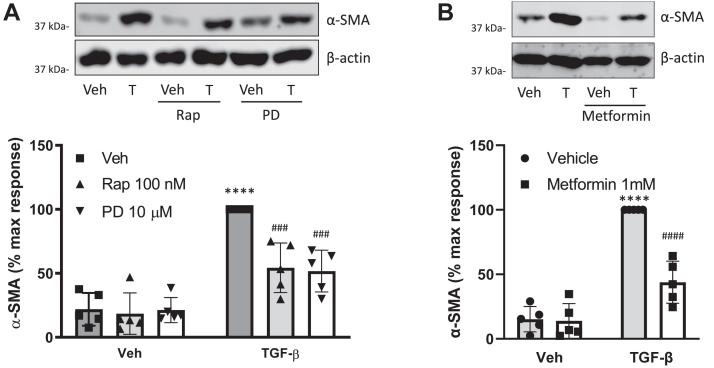
**mTORC1 inhibitor rapamycin and AMPK activator metformin inhibit TGF-β-induced alpha smooth muscleactinexpression.***A* and *B*, cells were stimulated for 3 days with TGF-β (1 ng/ml) in the presence or absence of (*A*) PD 102807 (10 μM) and rapamycin (100 nM) or (*B*) metformin (1 mM). Smooth muscle alpha-actin (α-SMA) expression was determined by immunoblotting. Representative blots are shown. Loading was corrected for β-actin. Data are means ± SD. ∗∗∗∗*p* < 0.0001 *versus* vehicle stimulation, ###*p* < 0.001, ####*p* < 0.0001 *versus* respective vehicle pretreated, stimulated condition; one-way ANOVA followed by Bonferroni multiple comparison test. AMPK, AMP-activated protein kinase; mTORC1, the mammalian target of rapamycin complex 1.

Our recently published study identified PD 102807 as a regulator of the TGF-β-induced ASM hypercontractile phenotype that is characterized by increased expression of smooth muscle α-actin (α-SMA). To determine if inhibition of mTORC1 can affect TGF-β-induced α-SMA expression, we treated ASM cells with TGF-β in the presence or absence of PD 102807 for 3 days; the mTORC1 inhibitor rapamycin was used a positive control. Both rapamycin and PD 102807 inhibited TGF-β-induced α-SMA expression to a similar degree (∼55% inhibition) (Fig. 6*A*). To determine if AMPK activation alone is sufficient to inhibit TGF-β-induced α-SMA expression, we treated cells with TGF-β in the presence or absence of the AMPK activator metformin for 3 days. Metformin inhibited TGF-β-induced α-SMA expression (Fig. 6*B*) to a similar degree as PD 102807 or rapamycin. These data further support a role for AMPK in regulation the mTORC1 pathway by PD 102807 to inhibit TGF-β-induced ASM phenotypic modulation.

## Discussion

mAChRs play a pivotal role in the physiological and pathological regulation of lung functions, and their antagonists are used as therapeutic agents in the treatment of asthma and COPD. Recent advances in GPCR pharmacology have laid the foundation for employing biased ligands to fine tune specific receptor signaling and function in target tissues. In this context, we recently demonstrated that PD 102807 is a biased M3 mAChR ligand that promotes arrestin recruitment to the M3 mAChR and induces GRK/arrestin-dependent AMPK phosphorylation. Most importantly, PD 102807 treatment inhibited development of the hypercontractile phenotype in human ASM cells. These studies, for the first time, characterized a biased M3 ligand and demonstrated feasibility of developing therapeutic agents based on biased agonism with previously unknown effector protein(s) in obstructive airway diseases. The discovery is particularly valuable because there are very few published studies on M3 mAChR biased signaling (30, 31, 32) as the field has been hindered by a lack of a biased ligand, until now.

**Figure 7:**
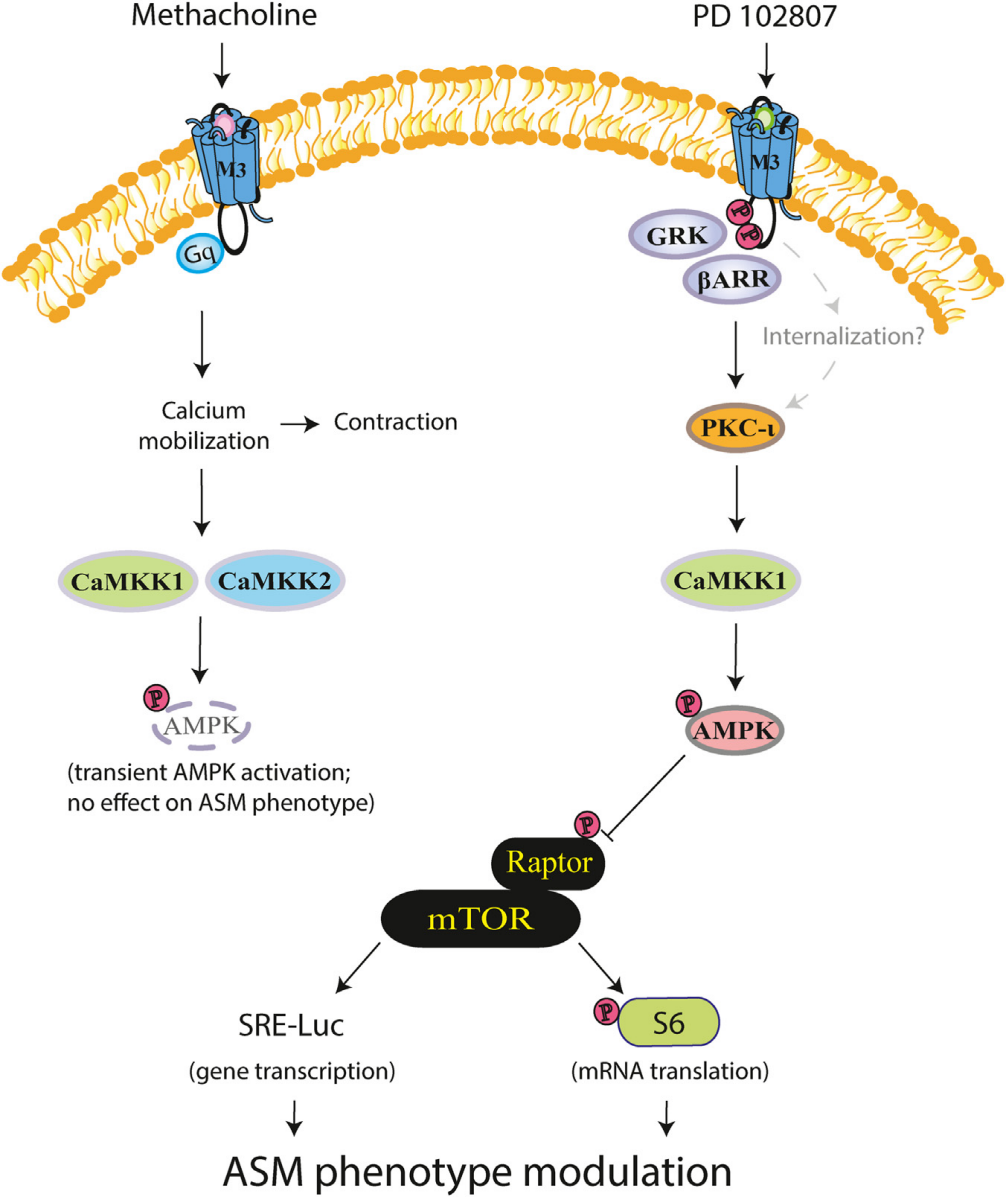
**PD 102807 is a biased M3 mAChR ligand that promotes GRK2/3-arrestin-dependent signaling *via* PKC-ι and CaMKK1.** Biased M3 mAChR signaling is mediated by PKC-ι, a member of the atypical family of PKC, and the AMPK kinase, CaMKK1. AMPK then phosphorylates Raptor to inhibit the mTORC1 complex, resulting in a decrease of both phosphorylation of S6 ribosomal protein (mRNA translation pathway) and SRE-Luc activity (gene transcription pathway), leading to inhibition of TGF-β-induced a-SMA expression, a feature of ASM phenotypic modulation to a contractile ASM phenotype. MCh induces a canonical Gq/calcium-dependent M3 mAChR signaling. Though not addressed in the current study, internalization may play a role in biased M3 mAChR signaling. MCh-induced AMPK phosphorylation is transient, dependent on calcium mobilization, and mediated by both CaMKK1 and CaMKK2 isoforms and independent of PKC-ι. Due to its transient nature, MCh-induced activation of AMPK does not modulate ASM phenotype. AMPK, AMP-activated protein kinase; ASM, airway smooth muscle; CaMKK, Ca^2+^/calmodulin-dependent kinase kinases; mAChR, muscarinic acetylcholine receptor; MCh, methacholine; PKC-ι, protein kinase C iota.

Our previous study characterized PD 102807 as a GRK/arrestin-biased mAChR ligand with AMPK as a downstream effector protein (22). The signaling mechanisms by which AMPK is activated or how it inhibits TGF-β-induced α-actin expression in ASM cells by PD 102807, were not established. In our current study we demonstrate that PD 102807-mediated AMPK α1 activation and downstream signaling is qualitatively distinct, and independent, from that of balanced MCh-stimulated signaling (Fig. 7).

### Differential regulation of AMPK phosphorylation by mAChR ligands

Our data indicate that both MCh and PD 102807 activate AMPK in ASM cells. CaMKKs are the only AMPK kinases involved in M3 mAChR-mediated AMPK phosphorylation. STK11 knockdown paradoxically increased PD 102807-induced p-AMPK, suggesting alternative AMPK activation pathways might be primed under these conditions. Even though both PD 102807- and MCh-induced p-AMPK are CaMKK-dependent, PD 102807-induced p-AMPK is mediated by CaMKK1, whereas MCh-induced p-AMPK is mediated by both CaMKK1 and CaMKK2 isoforms. This selective involvement of CaMKK1 in the biased M3 mAChR signaling is indicative of distinct regulatory mechanisms for the two CaMKK isoforms.

CaMKKs have been implicated in GPCR-induced AMPK phosphorylation (33, 34, 35), including that by M3 mAChRs (36), opioid receptors (37), and protease-activated receptor 1 (38). This GPCR-mediated activation of AMPK is specifically mediated by canonical Gq signaling and calcium mobilization (36, 37, 39) and largely dependent on the CaMKK2 isoform (33, 35). The only study so far to link arrestins and CaMKKs demonstrated that in adipocytes, β-arrestin 2 can associate with CaMKK2 to inhibit protease-activated receptor 2-induced AMPK activity (40). This finding is in agreement with our previous studies showing that β-arrestin 2 knockdown increases PD 102807-induced p-AMPK (22). While we are not aware of any other published studies on interactions between arrestins and CaMKKs, several studies have described interactions between β-arrestins and CaMKII—another member of the CaM-dependent kinase family, but one that does not phosphorylate AMPK and has functions that are distinct from those of CaMKKs (41, 42). Data from a variety of cell, tissue, and animal models demonstrates that β-arrestins promote CaMKII activation (43, 44, 45), by various mechanisms, including direct association with CaMKII as well as by facilitating formation of larger signaling complexes that include GCPRs. It is currently not known if similar mechanisms play a role in the regulation of CaMKK1 by β-arrestins.

CaMKK activity is enhanced by an increase in intracellular calcium concentrations—this facilitates calmodulin binding to CaMKKs—but both CaMKK isoforms can phosphorylate AMPK under calcium-free conditions (33) and can also exhibit kinase activity in the absence of calcium–calmodulin binding (46, 47, 48).

### PKC-ι is involved in biased M3 mAChR signaling

Another major finding of the current study is that the atypical isoform PKC-ι regulates biased M3 mAChR signaling. Unlike the classical and the novel PKC isoforms, the atypical PKCs (ζ and ι) are not activated by calcium, diacylglycerol, or phorbol esters (49). This is consistent with our findings that PD 102807-induced signaling is independent of Gq protein or calcium mobilization. Interestingly, the other atypical PKC isoform, PKCζ, has been shown to activate AMPK *via* STK11 (LKB1) phosphorylation (50, 51), suggesting that the atypical PKCs may have a class effect in regulating AMPK activity *via* multiple pathways. The link between GRK/arrestin and PKC-ι has not yet been found, and therefore, it remains unknown how PKC-ι is activated by biased M3 mAChR signaling. Activation of PKC-ι has not been fully elucidated and appears to be more complex than other PKC family members. Protein–protein interactions as well as various lipid mediators, including phosphatidylinositol (3,4,5)-trisphosphate generated by PI3K, have been shown to modulate activity of PKC-ι (27). While biased signaling is often considered to be mediated by the scaffolding function of arrestin proteins, the precise activation mechanisms of the various signaling molecules are still not well defined. Although further studies are needed to address the precise signaling mechanisms, our study represents a major step forward by identifying multiple mediators in the signaling cascade which leads to activation of AMPK in ASM cells. Further, one previous study indicated that protein kinase D (PKD) mediates arrestin-dependent M3 signaling (31). PKD can be activated by PKC, to which it is related structurally (52, 53). If PD 102807-mediated activation of AMPK also involves PKD remains to be determined.

Another potential mechanism of biased signaling that remains to be explored is receptor internalization. It was initially thought that GPCR activation only occurred at the plasma membrane, but more recent studies have shown that internalized receptors can contribute to signaling ((54, 55, 56, 57) and reviewed in (58)). When receptors internalize in endosomes, they may do so together with molecular machinery required for signaling, including G protein α subunit and second messenger-generating enzymes, as well as arrestins (58). Alternatively, GPCR activation can promote internalization of β-arrestins to promote signaling independent of GPCR internalization. In this scenario, β-arrestin briefly interacts with the activated GCPR but instead of forming a stable complex, it dissociates and then localizes to clathrin-coated vesicles without the GPCR (55, 56). We have previously identified GRK2/3 and β-arrestin 1 as crucial mediators of biased M3 mAChR signaling (22). Since GRK and arrestins both promote GPCR internalization, it is possible that GPCR/arrestin internalization contributes to PD 102807-induced signaling. However, while M3 mAChRs do internalize (59, 60, 61, 62), internalization has not yet been established as a *signaling* mechanism for these receptors. Future studies should address the potential contribution of GPCR internalization to biased M3 mAChR signaling.

### Signaling specificity downstream of AMPK

Different catalytic α subunit isoforms of AMPK may have distinct roles in regulating cell functions, including in ASM cells (63). We determined that both PD 102807- and MCh-induced AMPK activation involves AMPK α1 isoform. Further, we demonstrate that PD 102807-induced activation of AMPK α1 is associated with modulation of downstream signaling involving ACC and mTORC1. AMPK is an established negative regulator of mTOR pathways, in particular the mTORC1, a multisubunit complex which includes mTOR and regulatory-associated protein of mTOR (Raptor) (28). mTORC1 is a crucial regulator of protein synthesis that has previously been shown to mediate contractile protein accumulation in ASM cells (64). The AMPK-mediated phosphorylation of Raptor is the key step in inhibiting mTORC1 function and is mediated by AMPK α1 (but not α2). This finding is further corroborated by PD 102807 inhibiting SRE-driven luciferase expression and phosphorylation of S6 ribosomal protein; both regulated by mTOR (29). In addition, we show that these findings hold true for other cell types; PD 102807 induces M3 mAChR-dependent AMPK phosphorylation in HEK 293 cells and similarly inhibits S6 protein phosphorylation and SRE-Luc activity.

### Functional effect of PD 102807

Our current and previous studies suggest that treatment of ASM cells with PD 102807 inhibits TGF-β-induced α-smooth muscle actin expression. The unbiased agonist MCh does not abrogate α-smooth muscle actin expression, and in fact, other studies have suggested that MCh promotes contractile protein expression (65). Furthermore, the mTOR inhibitor rapamycin or the AMPK activator metformin resulted in inhibition of TGF-β-induced α-SMA expression similar to that effected by PD 102807, providing further evidence for the role of the AMPK-mTORC1 axis in inhibition of TGF-β-induced ASM cell remodeling. The finding that both MCh and PD 102807 induce AMPK activation, but with different functional outcomes, raises the question as to how the two signaling pathways are different. While both biased and canonical M3 signaling share AMPK α1 as the common signaling molecule, the interplay of (1) different kinases phosphorylating AMPK (CaMKK1 for biased signaling *versus* CaMKK1 and CaMKK2 for canonical signaling) and (2) the difference in the kinetic profile of AMPK phosphorylation (slow, sustained for biased signaling; fast on, fast off for canonical signaling (22)) likely underlies the differences in functional outcomes. Sustained AMPK activation is likely required for regulating phenotypic modulation of ASM, as this takes place over a prolonged period. In addition, MCh-induced Gq signaling may also promote functions that oppose beneficial effects of AMPK activation.

In summary, our study demonstrates distinct signaling mechanisms activated by M3 mAChR ligands in ASM cells eliciting unique functional outcomes. Skewing M3 mAChR away from calcium mobilization but toward AMPK activation could have therapeutic benefit in obstructive airway diseases. Our studies indicate that PD 102807 may have beneficial effects on airway remodeling and that biased M3 mAChR signaling has therapeutic potential for this hard-to-treat feature of obstructive airways diseases.

## Experimental procedures

### Reagents

Reagents used throughout this study were obtained from the following sources: PD 102807, and STO-609 acetate, (Bio-Techne), acetyl-β-methylcholine chloride, and 5z-7-oxozealenol (Millipore), Gö 6983 (Selleck Chemicals), PKC-iota inhibitor 1 (compound 19) (MedChemExpress), and metformin (Cayman Chem). All other reagents described in the study were purchased from Sigma, ThermoFisher, or sources described previously (66).

### Cell culture

Human ASM cells stably expressing telomerase reverse transcriptase (hTERT) which maintain physiological levels of expression of M3 mAChR (22, 67) were provided by Dr W.T Gerthoffer. A plasmid containing hTERT cDNA expression vector was transduced into primary ASM cells by retrovirus. Expression of hTERT serves to prevent rapid cell senescence and loss of M3 mAChR signaling which occurs in in isolated ASM cells. These cells maintain ASM spindle-shape cell morphology, GPCR-responsiveness, and contractile protein expression, as well as contractility. Telomerase reverse transcriptase expression was maintained using G418 (500 μg/ml; Millipore). Cultures were maintained in Ham’s F12 media supplemented with 10% fetal bovine serum; 24 h prior to experiments, cells were serum starved as described previously (22).

HEK 293 cells were maintained in Dulbecco’s modified Eagle’s media supplemented with 10% fetal bovine serum and serum deprived before stimulation, as described previously (22).

### Small interfering RNA-mediated knockdown

siRNA On-Targetplus Smartpool oligos directed against CaMKK1 (cat. No. L-004912), CaMKK2 (cat. No. L-004842), STK11 (cat. No. L-005035), PKC-ι (cat. No. L-004656), and Dharmafect 1 were purchased from Dharmacon. GRK2/3 siRNA oligos (5′-GAT CTT CGA CTC ATA CAT CTT-3′) were annealed at 95 °C for 5 min and allowed to cool to room temperature. 5 μg of oligos were used to transfect human ASM cells, using Dharmafect 1 as per manufacturer’s instructions, with mock transfection control consisting of Dharmafect 1 without oligos. Twenty-four hours after transfection, the cells were replated for subsequent assessment of protein expression.

### Immunoblot analysis

Cells were serum-deprived for 24 h, refed, and then stimulated for the indicated durations. Cells were washed with ice-cold PBS, then lysed with 2x Laemmli buffer (50 mM pH 6.8 Tris solution, 10% glycerol, 2% SDS, 0.01% bromophenol blue, and 2.5% beta-ME). Lysates were loaded and separated on either 10% Tris-glycine polyacrylamide gels or 4 to 12% gradient precast Novus Tris-glycine polyacrylamide gels (Invitrogen) according to the manufacturer’s instructions. Blots were probed with specific antibodies directed against p-AMPK, total AMPK, AMPK α1, AMPK α2, p-ACC, LKB1 (SKT11), CaMKK2, PKC-ι, p-mTOR, total mTOR, p-Raptor, p-S6 ribosomal protein, α-SMA (Cell Signaling Technology), CaMKK1 (Novus Biologicals), and β-actin (Millipore Sigma).

### ASM cell permeabilization and agonist stimulation

Cells were plated onto a 12-well plate, allowed to attach overnight, and growth arrested for 24 h. Prior to stimulation, cells were permeabilized with 100 nM β-escin (Millipore) (68) for 5 min at room temperature in calcium-free Hank’s Balanced Salt Solution (HBSS)(Fisher Scientific). Performing a short treatment with a low concentration of β-escin renders cells permeable to monovalent and divalent ions; treatment with micromolar concentrations in excess of 20 min may lead to cell membrane permeability to larger molecules (69). To remove excess β-escin and calcium ions, cells were washed twice with calcium-free HBSS. Wells were refreshed with calcium-free HBSS, and then, various concentrations of calcium were reintroduced into the wells by adding calcium chloride and equilibrating for 5 min. Cells were then treated with vehicle, PD 102807 (100 μM; 20 min), or MCh (100 μM; 5 min) and lysed for immunoblot analysis.

### Intracellular calcium analysis

Human ASM cells were plated onto 96-well plates and loaded with 2 μM Fluo-4 acetoxymethyl (BD Biosciences) and probenecid for 1 h according to manufacturer’s instructions. Indicated final concentrations of agonists were added by an automated pipetting system [FlexStation 3 (Molecular Devices)] in duplicate. The calcium-bound Fluo-4 dye fluorescence emission was detected at 525 nm using excitation wavelength of 485 nm by Flexstation 3 and SoftMax Pro 7 software (Molecular Devices), as described previously (22). Measurements were taken every 2 s up to 25 min; basal fluorescence was measured for 2 min before the stimuli were added to the wells. The net peak Ca^2+^ response was calculated as [(Agonist-induced fluorescence units) – (Basal fluorescence units)]. Maximal Ca^2+^ increase was induced by stimulating cells with the calcium ionophore ionomycin (1 μM) and was used to normalize agonist-induced Ca^2+^ response in each experiment.

### Luciferase activity assay

Stable expression of a reporter construct for SRE-luciferase was established in hTERT ASM and HEK 293 cells using Cignal Lenti luciferase reporter viral particles (SA Biosciences). Expression of SRE-Luc was maintained using puromycin (2 μg/ml). Cells were serum-deprived for 8 h and then stimulated for 18 h and harvested in passive lysis buffer. Luminescence was determined after adding firefly luciferase substrate reagent using a microplate luminometer.

### Statistical analysis

Data analysis was performed using GraphPad (GraphPad Software), and data are expressed as mean ± SD. Group comparisons were performed using a one-way ANOVA followed by Bonferroni’s multiple comparison test. Two-tailed *t* test was used where appropriate, for comparisons of means of two groups. A value of *p* < 0.05 was considered significant to reject the null hypothesis.

## Data availability

All the data are contained within the manuscript.

## Supporting information

This article contains supporting information.

## Conflict of interest

The authors declare that they have no conflicts of interest with the contents of this article.

## References

[1] Gross N.J., Skorodin M.S. (1984). Role of the parasympathetic system in airway obstruction due to emphysema. N. Engl. J. Med..

[2] Bos I.S., Gosens R., Zuidhof A.B., Schaafsma D., Halayko A.J., Meurs H. (2007). Inhibition of allergen-induced airway remodelling by tiotropium and budesonide: a comparison. Eur. Respir. J..

[3] Kistemaker L.E., Bos I.S., Hylkema M.N., Nawijn M.C., Hiemstra P.S., Wess J. (2013). Muscarinic receptor subtype-specific effects on cigarette smoke-induced inflammation in mice. Eur. Respir. J..

[4] Pera T., Zuidhof A., Valadas J., Smit M., Schoemaker R.G., Gosens R. (2011). Tiotropium inhibits pulmonary inflammation and remodelling in a Guinea pig model of copd. Eur. Respir. J..

[5] Gosens R., Bos I.S., Zaagsma J., Meurs H. (2005). Protective effects of tiotropium bromide in the progression of airway smooth muscle remodeling. Am. J. Respir. Crit. Care Med..

[6] Benovic J.L., Kuhn H., Weyand I., Codina J., Caron M.G., Lefkowitz R.J. (1987). Functional desensitization of the isolated beta-adrenergic receptor by the beta-adrenergic receptor kinase: potential role of an analog of the retinal protein arrestin (48-kda protein). Proc. Natl. Acad. Sci. U. S. A..

[7] Lohse M.J., Benovic J.L., Codina J., Caron M.G., Lefkowitz R.J. (1990). Beta-arrestin: a protein that regulates beta-adrenergic receptor function. Science.

[8] Kim J., Ahn S., Ren X.R., Whalen E.J., Reiter E., Wei H. (2005). Functional antagonism of different g protein-coupled receptor kinases for beta-arrestin-mediated angiotensin ii receptor signaling. Proc. Natl. Acad. Sci. U. S. A..

[9] Ren X.R., Reiter E., Ahn S., Kim J., Chen W., Lefkowitz R.J. (2005). Different g protein-coupled receptor kinases govern g protein and beta-arrestin-mediated signaling of v2 vasopressin receptor. Proc. Natl. Acad. Sci. U. S. A..

[10] Smith J.S., Lefkowitz R.J., Rajagopal S. (2018). Biased signalling: from simple switches to allosteric microprocessors. Nat. Rev. Drug Discov..

[11] Kim I.M., Tilley D.G., Chen J., Salazar N.C., Whalen E.J., Violin J.D. (2008). Beta-blockers alprenolol and carvedilol stimulate beta-arrestin-mediated egfr transactivation. Proc. Natl. Acad. Sci. U. S. A..

[12] Patel P.A., Tilley D.G., Rockman H.A. (2008). Beta-arrestin-mediated signaling in the heart. Circ. J..

[13] Forkuo G.S., Kim H., Thanawala V.J., Al-Sawalha N., Valdez D., Joshi R. (2016). Phosphodiesterase 4 inhibitors attenuate the asthma phenotype produced by beta2-adrenoceptor agonists in phenylethanolamine n-methyltransferase-knockout mice. Am. J. Respir. Cell Mol. Biol..

[14] Penn R.B., Bond R.A., Walker J.K. (2014). Gpcrs and arrestins in airways: implications for asthma. Handb Exp. Pharmacol..

[15] Walker J.K., Penn R.B., Hanania N.A., Dickey B.F., Bond R.A. (2011). New perspectives regarding beta(2) -adrenoceptor ligands in the treatment of asthma. Br. J. Pharmacol..

[16] Nguyen L.P., Al-Sawalha N.A., Parra S., Pokkunuri I., Omoluabi O., Okulate A.A. (2017). Beta2-adrenoceptor signaling in airway epithelial cells promotes eosinophilic inflammation, mucous metaplasia, and airway contractility. Proc. Natl. Acad. Sci. U. S. A..

[17] De Pascali F., Ippolito M., Wolfe E., Komolov K.E., Hopfinger N., Lemenze D. (2022). Beta(2) -adrenoceptor agonist profiling reveals biased signalling phenotypes for the beta(2) -adrenoceptor with possible implications for the treatment of asthma. Br. J. Pharmacol..

[18] Panettieri R.A., Pera T., Liggett S.B., Benovic J.L., Penn R.B. (2018). Pepducins as a potential treatment strategy for asthma and copd. Curr. Opin. Pharmacol..

[19] Shah S.D., Lind C., De Pascali F., Penn R.B., MacKerell A.D., Deshpande D.A. (2022). In silico identification of a beta(2)-adrenoceptor allosteric site that selectively augments canonical beta(2)ar-gs signaling and function. Proc. Natl. Acad. Sci. U. S. A..

[20] Olianas M.C., Onali P. (1999). Pd 102807, a novel muscarinic m4 receptor antagonist, discriminates between striatal and cortical muscarinic receptors coupled to cyclic amp. Life Sci..

[21] Augelli-Szafran C.E., Jaen J.C., Moreland D.W., Nelson C.B., Penvose-Yi J.R., Schwarz R.D. (1998). Identification and characterization of m4 selective muscarinic antagonists. Bioorg. Med. Chem. Lett..

[22] Tompkins E., Mimic B., Cuevas-Mora K., Schorsch H., Shah S.D., Deshpande D.A. (2022). Pd 102807 induces m3 machr-dependent grk-/arrestin-biased signaling in airway smooth muscle cells. Am. J. Respir. Cell Mol. Biol..

[23] Hardie D.G., Ross F.A., Hawley S.A. (2012). Ampk: a nutrient and energy sensor that maintains energy homeostasis. Nat. Rev. Mol. Cell Biol..

[24] Jeon S.M. (2016). Regulation and function of ampk in physiology and diseases. Exp. Mol. Med..

[25] Billington C.K., Penn R.B. (2003). Signaling and regulation of g protein-coupled receptors in airway smooth muscle. Respir. Res..

[26] Pitcher J., Lohse M.J., Codina J., Caron M.G., Lefkowitz R.J. (1992). Desensitization of the isolated beta 2-adrenergic receptor by beta-adrenergic receptor kinase, camp-dependent protein kinase, and protein kinase c occurs via distinct molecular mechanisms. Biochemistry.

[27] Suzuki A., Akimoto K., Ohno S. (2003). Protein kinase c lambda/iota (pkclambda/iota): a pkc isotype essential for the development of multicellular organisms. J. Biochem..

[28] Proud C.G. (2004). Role of mtor signalling in the control of translation initiation and elongation by nutrients. Curr. Top Microbiol. Immunol..

[29] Hernandez-Lara M.A., Yadav S.K., Shah S.D., Okumura M., Yokoyama Y., Penn R.B. (2022). Regulation of airway smooth muscle cell proliferation by diacylglycerol kinase: relevance to airway remodeling in asthma. Int. J. Mol. Sci..

[30] Bradley S.J., Wiegman C.H., Iglesias M.M., Kong K.C., Butcher A.J., Plouffe B. (2016). Mapping physiological g protein-coupled receptor signaling pathways reveals a role for receptor phosphorylation in airway contraction. Proc. Natl. Acad. Sci. U. S. A..

[31] Kong K.C., Butcher A.J., McWilliams P., Jones D., Wess J., Hamdan F.F. (2010). M3-muscarinic receptor promotes insulin release via receptor phosphorylation/arrestin-dependent activation of protein kinase d1. Proc. Natl. Acad. Sci. U. S. A..

[32] Poulin B., Butcher A., McWilliams P., Bourgognon J.M., Pawlak R., Kong K.C. (2010). The m3-muscarinic receptor regulates learning and memory in a receptor phosphorylation/arrestin-dependent manner. Proc. Natl. Acad. Sci. U. S. A..

[33] Hawley S.A., Pan D.A., Mustard K.J., Ross L., Bain J., Edelman A.M. (2005). Calmodulin-dependent protein kinase kinase-beta is an alternative upstream kinase for amp-activated protein kinase. Cell Metab..

[34] Hurley R.L., Anderson K.A., Franzone J.M., Kemp B.E., Means A.R., Witters L.A. (2005). The ca2+/calmodulin-dependent protein kinase kinases are amp-activated protein kinase kinases. J. Biol. Chem..

[35] Woods A., Dickerson K., Heath R., Hong S.P., Momcilovic M., Johnstone S.R. (2005). Ca2+/calmodulin-dependent protein kinase kinase-beta acts upstream of amp-activated protein kinase in mammalian cells. Cell Metab..

[36] Merlin J., Evans B.A., Csikasz R.I., Bengtsson T., Summers R.J., Hutchinson D.S. (2010). The m3-muscarinic acetylcholine receptor stimulates glucose uptake in l6 skeletal muscle cells by a camkk-ampk-dependent mechanism. Cell. Signal..

[37] Olianas M.C., Dedoni S., Olianas A., Onali P. (2012). Delta-opioid receptors stimulate the metabolic sensor amp-activated protein kinase through coincident signaling with g(q/11)-coupled receptors. Mol. Pharmacol..

[38] Stahmann N., Woods A., Carling D., Heller R. (2006). Thrombin activates amp-activated protein kinase in endothelial cells via a pathway involving ca2+/calmodulin-dependent protein kinase kinase beta. Mol. Cell. Biol..

[39] Kishi K., Yuasa T., Minami A., Yamada M., Hagi A., Hayashi H. (2000). Amp-activated protein kinase is activated by the stimulations of g(q)-coupled receptors. Biochem. Biophys. Res. Commun..

[40] Wang P., Jiang Y., Wang Y., Shyy J.Y., DeFea K.A. (2010). Beta-arrestin inhibits camkkbeta-dependent ampk activation downstream of protease-activated-receptor-2. BMC Biochem..

[41] Means A.R. (2000). Regulatory cascades involving calmodulin-dependent protein kinases. Mol. Endocrinol..

[42] Takemoto-Kimura S., Suzuki K., Horigane S.I., Kamijo S., Inoue M., Sakamoto M. (2017). Calmodulin kinases: essential regulators in health and disease. J. Neurochem..

[43] Mangmool S., Shukla A.K., Rockman H.A. (2010). Beta-arrestin-dependent activation of ca(2+)/calmodulin kinase ii after beta(1)-adrenergic receptor stimulation. J. Cell Biol..

[44] Xu B., Li M., Wang Y., Zhao M., Morotti S., Shi Q. (2020). Grk5 controls sap97-dependent cardiotoxic beta(1) adrenergic receptor-camkii signaling in heart failure. Circ. Res..

[45] Zhu L., Almaca J., Dadi P.K., Hong H., Sakamoto W., Rossi M. (2017). Beta-arrestin-2 is an essential regulator of pancreatic beta-cell function under physiological and pathophysiological conditions. Nat. Commun..

[46] Edelman A.M., Mitchelhill K.I., Selbert M.A., Anderson K.A., Hook S.S., Stapleton D. (1996). Multiple ca(2+)-calmodulin-dependent protein kinase kinases from rat brain. Purification, regulation by ca(2+)-calmodulin, and partial amino acid sequence. J. Biol. Chem..

[47] Haribabu B., Hook S.S., Selbert M.A., Goldstein E.G., Tomhave E.D., Edelman A.M. (1995). Human calcium-calmodulin dependent protein kinase i: Cdna cloning, domain structure and activation by phosphorylation at threonine-177 by calcium-calmodulin dependent protein kinase I kinase. EMBO J..

[48] Anderson K.A., Means R.L., Huang Q.H., Kemp B.E., Goldstein E.G., Selbert M.A. (1998). Components of a calmodulin-dependent protein kinase cascade. Molecular cloning, functional characterization and cellular localization of ca2+/calmodulin-dependent protein kinase kinase beta. J. Biol. Chem..

[49] Moscat J., Diaz-Meco M.T. (2000). The atypical protein kinase cs. Functional specificity mediated by specific protein adapters. EMBO Rep..

[50] Xie Z., Dong Y., Scholz R., Neumann D., Zou M.H. (2008). Phosphorylation of lkb1 at serine 428 by protein kinase c-zeta is required for metformin-enhanced activation of the amp-activated protein kinase in endothelial cells. Circulation.

[51] Xie Z., Dong Y., Zhang J., Scholz R., Neumann D., Zou M.H. (2009). Identification of the serine 307 of lkb1 as a novel phosphorylation site essential for its nucleocytoplasmic transport and endothelial cell angiogenesis. Mol. Cell. Biol..

[52] Steinberg S.F. (2021). Decoding the cardiac actions of protein kinase d isoforms. Mol. Pharmacol..

[53] Valverde A.M., Sinnett-Smith J., Van Lint J., Rozengurt E. (1994). Molecular cloning and characterization of protein kinase d: a target for diacylglycerol and phorbol esters with a distinctive catalytic domain. Proc. Natl. Acad. Sci. U. S. A..

[54] Beautrait A., Paradis J.S., Zimmerman B., Giubilaro J., Nikolajev L., Armando S. (2017). A new inhibitor of the beta-arrestin/ap2 endocytic complex reveals interplay between gpcr internalization and signalling. Nat. Commun..

[55] Eichel K., Jullie D., Barsi-Rhyne B., Latorraca N.R., Masureel M., Sibarita J.B. (2018). Catalytic activation of beta-arrestin by gpcrs. Nature.

[56] Eichel K., Jullie D., von Zastrow M. (2016). Beta-arrestin drives map kinase signalling from clathrin-coated structures after gpcr dissociation. Nat. Cell Biol..

[57] Luttrell L.M., Ferguson S.S., Daaka Y., Miller W.E., Maudsley S., Della Rocca G.J. (1999). Beta-arrestin-dependent formation of beta2 adrenergic receptor-src protein kinase complexes. Science.

[58] Wang W., Qiao Y., Li Z. (2018). New insights into modes of gpcr activation. Trends Pharmacol. Sci..

[59] Bhattacharya S., Mahavadi S., Al-Shboul O., Rajagopal S., Grider J.R., Murthy K.S. (2013). Differential regulation of muscarinic m2 and m3 receptor signaling in gastrointestinal smooth muscle by caveolin-1. Am. J. Physiol. Cell Physiol..

[60] Lee K.B., Pals-Rylaarsdam R., Benovic J.L., Hosey M.M. (1998). Arrestin-independent internalization of the m1, m3, and m4 subtypes of muscarinic cholinergic receptors. J. Biol. Chem..

[61] Sanchez-Fernandez G., Cabezudo S., Garcia-Hoz C., Tobin A.B., Mayor F., Ribas C. (2013). Erk5 activation by gq-coupled muscarinic receptors is independent of receptor internalization and beta-arrestin recruitment. PLoS One.

[62] Vogler O., Nolte B., Voss M., Schmidt M., Jakobs K.H., van Koppen C.J. (1999). Regulation of muscarinic acetylcholine receptor sequestration and function by beta-arrestin. J. Biol. Chem..

[63] Liu L., Pan Y., Song Y., Su X., Ke R., Yang L. (2016). Activation of ampk alpha2 inhibits airway smooth muscle cells proliferation. Eur. J. Pharmacol..

[64] Halayko A.J., Kartha S., Stelmack G.L., McConville J., Tam J., Camoretti-Mercado B. (2004). Phophatidylinositol-3 kinase/mammalian target of rapamycin/p70s6k regulates contractile protein accumulation in airway myocyte differentiation. Am. J. Respir. Cell Mol. Biol..

[65] Oenema T.A., Smit M., Smedinga L., Racke K., Halayko A.J., Meurs H. (2012). Muscarinic receptor stimulation augments tgf-beta1-induced contractile protein expression by airway smooth muscle cells. Am. J. Physiol. Lung Cell. Mol. Physiol..

[66] Pera T., Deshpande D.A., Ippolito M., Wang B., Gavrila A., Michael J.V. (2018). Biased signaling of the proton-sensing receptor ogr1 by benzodiazepines. FASEB J..

[67] Deshpande D.A., Yan H., Kong K.C., Tiegs B.C., Morgan S.J., Pera T. (2014). Exploiting functional domains of grk2/3 to alter the competitive balance of pro- and anticontractile signaling in airway smooth muscle. FASEB J..

[68] Kannan M.S., Prakash Y.S., Brenner T., Mickelson J.R., Sieck G.C. (1997). Role of ryanodine receptor channels in ca2+ oscillations of porcine tracheal smooth muscle. Am. J. Physiol..

[69] Morton M.J., Main M.J. (2013). Use of escin as a perforating agent on the ionworks quattro automated electrophysiology platform. J. Biomol. Screen..

