# Supplementary material for: The biased M3 mAChR ligand PD 102807 mediates qualitatively distinct signaling to regulate airway smooth muscle phenotype
Source: J Biol Chem. 2023 Sep 1;299(10):105209. doi: 10.1016/j.jbc.2023.105209 (PMC10520882; doi:10.1016/j.jbc.2023.105209)
Supplement: Supporting Figures S1–S3 [file mmc1.docx]

**Supporting Information**

The biased M3 mAChR ligand PD 102807 mediates qualitatively distinct signaling to regulate airway smooth muscle phenotype

Eric Tompkins, Bogdana Mimic, Raymond B. Penn and Tonio Pera

Center for Translational Medicine, Jane and Leonard Korman Respiratory Institute, Department of Medicine; Philadelphia, PA, USA


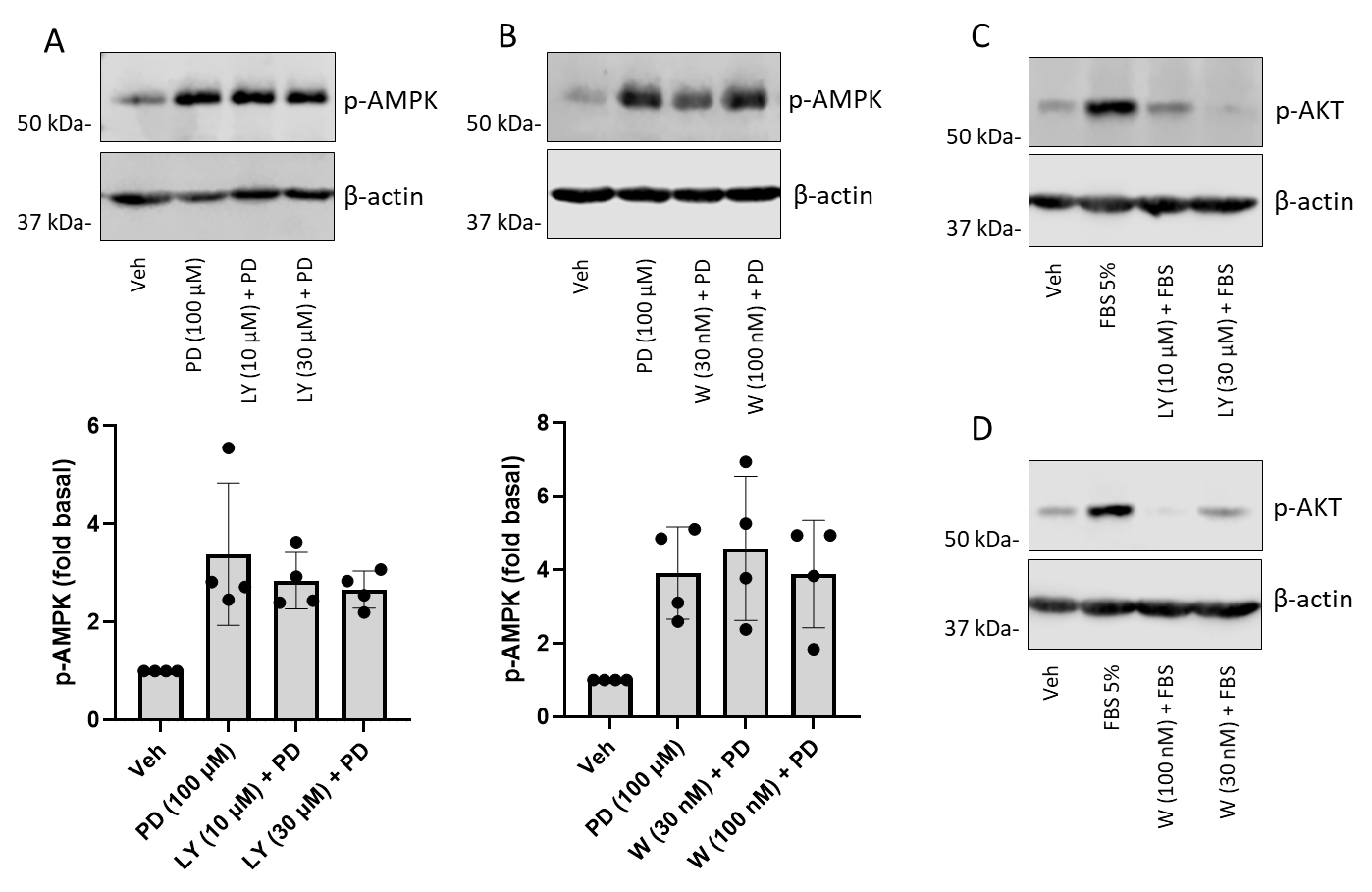


**Figure S1:** The PI3K inhibitors A) LY294002 (10 or 30 µM; 15 min preincubation) and B) wortmannin (30 and 100 nM; 15 min pre-incubation) do not inhibit PD 102807-induced (100 µM; 20 min) p-AMPK in ASM cells. Data are means +/- SD. Fetal bovine serum (FBS; 5%; 10 min)-induced p-AKT (positive control) is inhibited by C) LY294002 and D) wortmannin at both concentrations of inhibitors, in ASM cells. Representative images of 4 experiments are shown.


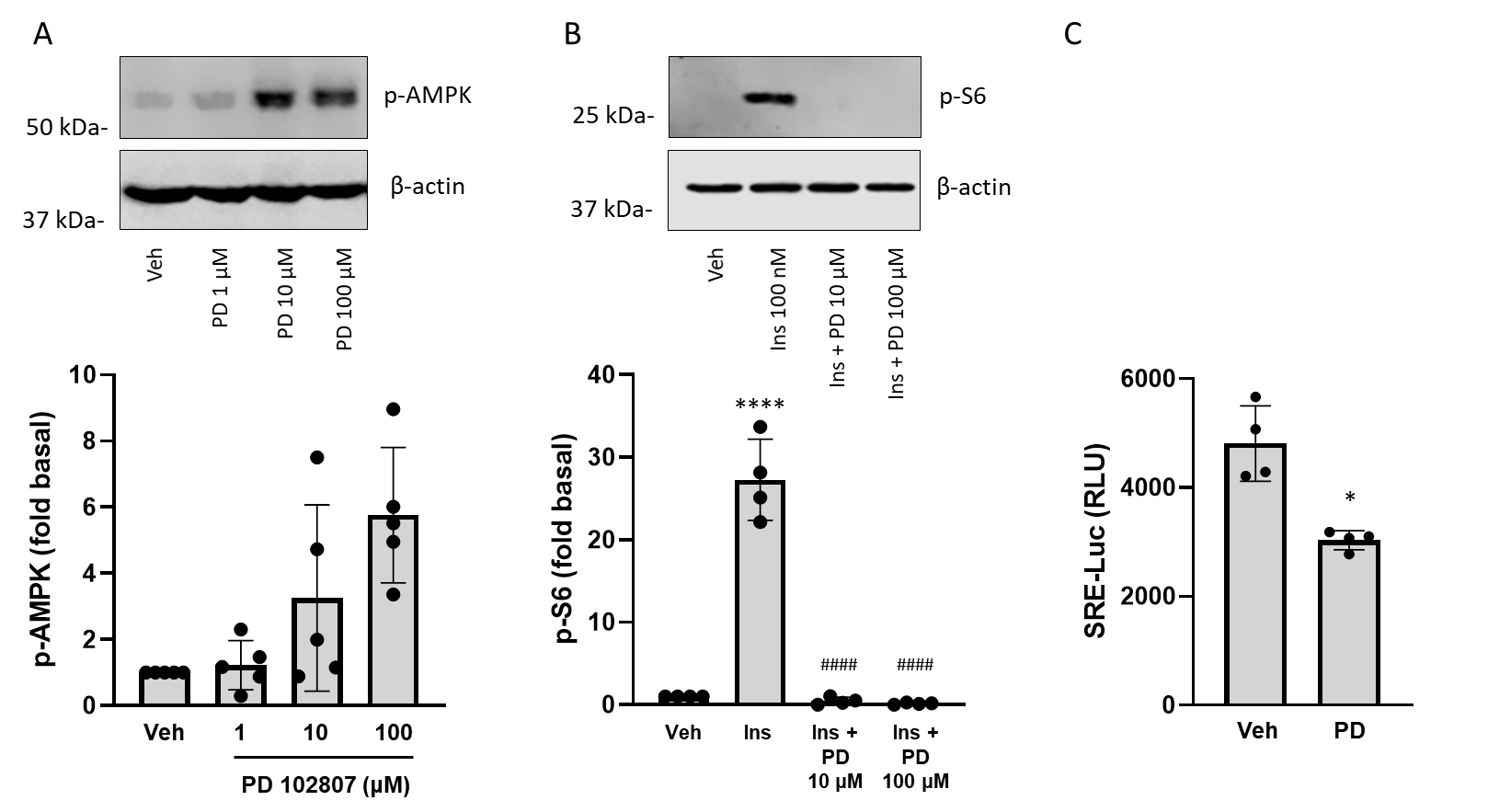


**Figure S2:** PD 102807 induces AMPK phosphorylation and inhibits mTOR signaling in HEK293 cells. A) AMPK phosphorylation induced by increasing concentrations of PD 102807 (1 - 100 μM; 20 min). B) PD 102807 (10 or 100 μM; 20 min pretreatment) inhibits insulin-induced (100 nM; 30 min) phosphorylation of S6 ribosomal protein. C) PD 102807 (10 μM; 18 h) inhibits basal SRE-Luc activity. Data are means ± SD. *p < 0.05, ****p < 0.0001 vs vehicle, ####p < 0.0001 vs respective vehicle pretreated, stimulated condition; one-way ANOVA followed by Bonferroni multiple comparison test; two-tailed Student’s t-test.


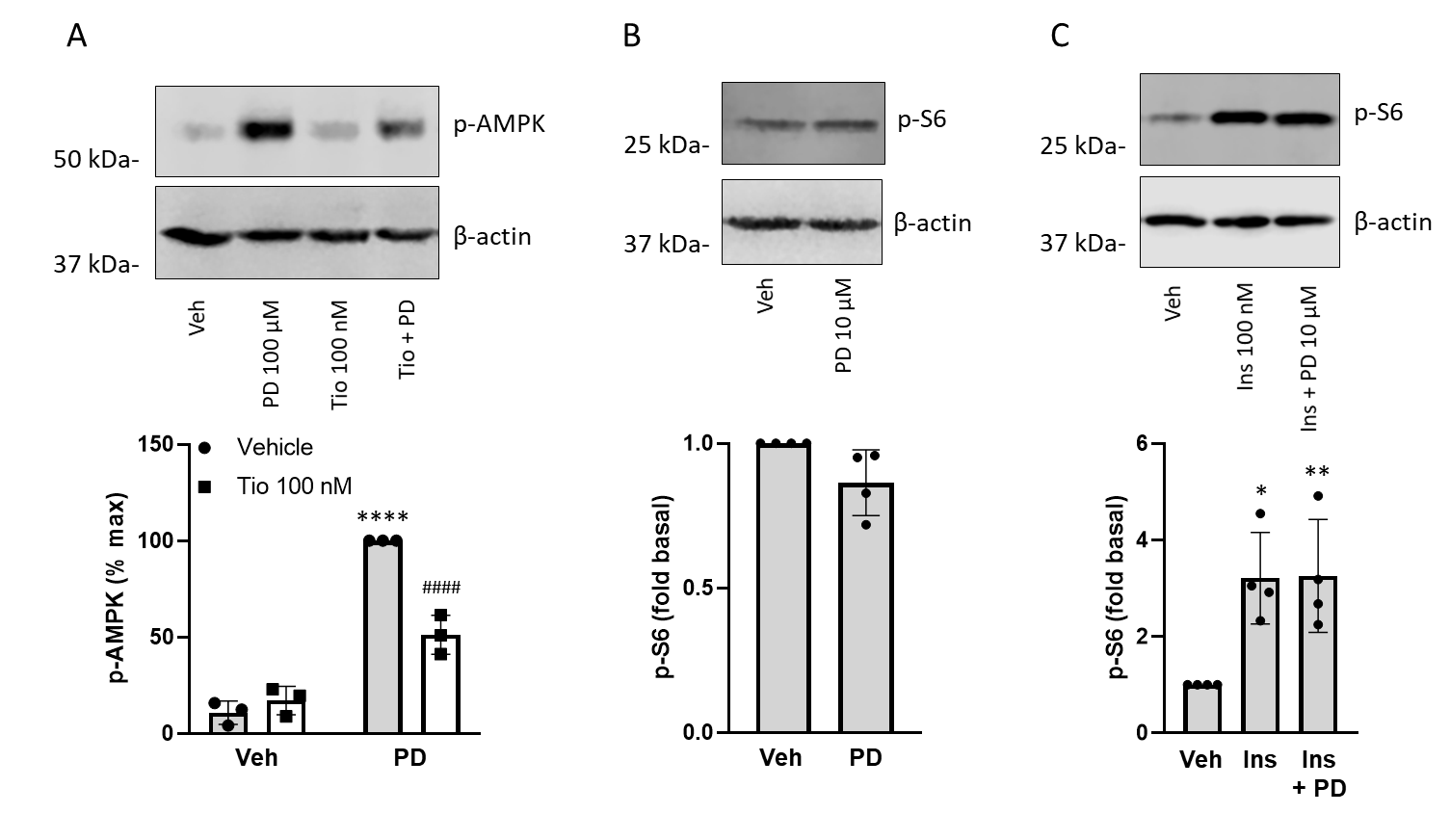


**Figure S3:** PD 102807-induced inhibition of mTOR is M3 mAChR dependent. A) PD 102807-induced (100 μM; 20 min) AMPK phosphorylation is inhibited by muscarinic antagonist tiotropium (100 nM: 30 min pretreatment) in HEK293 cells. B) Basal and C) insulin-induced (100 nM; 30 min) phosphorylation of S6 ribosomal protein is not affected by PD 102807 (10 μM; 20 min stimulation or pretreatment) in ASM cells which have no or low M3 mAChR expression. Data are means ± SD. *p < 0.05, **p < 0.01, ****p < 0.0001 vs vehicle, ####p < 0.0001 vs respective vehicle pretreated, stimulated condition; one-way ANOVA followed by Bonferroni multiple comparison test or Dunnett’s test.
